# Supplementary material for: Efficient Expression in Leishmania tarentolae (LEXSY) of the Receptor-Binding Domain of the SARS-CoV-2 S-Protein and the Acetylcholine-Binding Protein from Lymnaea stagnalis
Source: Molecules. 2024 Feb 21;29(5):943. doi: 10.3390/molecules29050943 (PMC10934791; doi:10.3390/molecules29050943)
Supplement: Supplementary file 1 [file molecules-29-00943-s001.zip › molecules-2761199-supplementary.pdf]

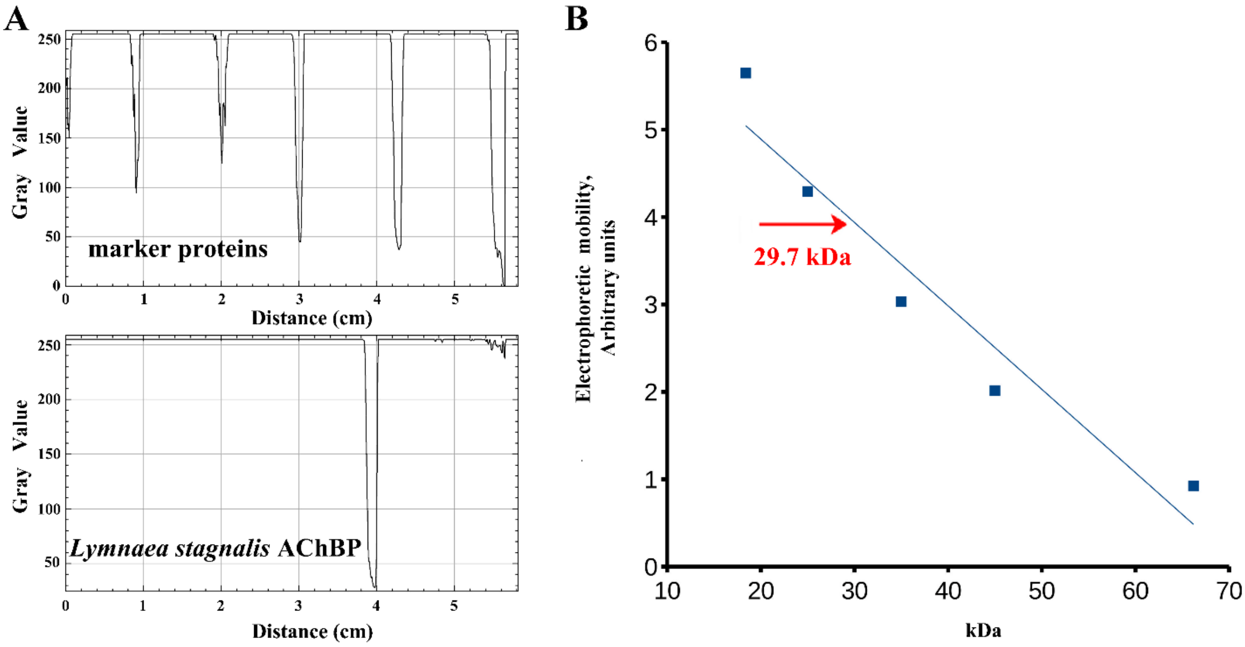

Figure S1. Calibration curve used to estimate the obtained product (monomeric form of *Lymnaea stagnalis* AChBP) molecular mass *via* electrophoretic mobility. The estimated mass was 29.7 kDa.
